# Automated Respiratory Rate Counter to Assess Children for Symptoms of Pneumonia: Protocol for Cross-Sectional Usability and Acceptability Studies in Ethiopia and Nepal

**DOI:** 10.2196/14405

**Published:** 2020-03-30

**Authors:** Kevin Baker, Alice Maurel, Charlotte Ward, Dawit Getachew, Tedila Habte, Cindy McWhorter, Paul LaBarre, Jonas Karlström, Max Petzold, Karin Källander

**Affiliations:** 1 Malaria Consortium London United Kingdom; 2 Karolinska Institute Stockholm Sweden; 3 Malaria Consortium Addis Ababa Ethiopia; 4 United Nations Children’s Fund Supply Division Copenhagen Denmark; 5 School of Public Health and Community Medicine Institute of Medicine University of Gothenburg Gothenburg Sweden; 6 University of the Witwatersrand Johannesburg South Africa; 7 United Nations Children’s Fund Programme Division New York, NY United States

**Keywords:** community health worker, pneumonia, child, respiratory rate, Nepal, Ethiopia

## Abstract

**Background:**

Manually counting a child’s respiratory rate (RR) for 60 seconds using an acute respiratory infection timer is the World Health Organization (WHO) recommended method for detecting fast breathing as a sign of pneumonia. However, counting the RR is challenging and misclassification of an observed rate is common, often leading to inappropriate treatment. To address this gap, the acute respiratory infection diagnostic aid (ARIDA) project was initiated in response to a call for better pneumonia diagnostic aids and aimed to identify and assess automated RR counters for classifying fast breathing pneumonia when used by front-line health workers in resource-limited community settings and health facilities. The Children’s Automated Respiration Monitor (ChARM), an automated RR diagnostic aid using accelerometer technology developed by Koninklijke Philips NV, and the Rad-G, a multimodal RR diagnostic and pulse oximeter developed by Masimo, were the two devices tested in these studies conducted in the Southern Nations, Nationalities, and Peoples’ Region in Ethiopia and in the Karnali region in Nepal.

**Objective:**

In these studies, we aimed to understand the usability of two new automated RR diagnostic aids for community health workers (CHWs; health extension workers [Ethiopia] and female community health volunteers [Nepal]) and their acceptability to CHWs in Ethiopia and Nepal, first-level health facility workers (FLHFWs) in Ethiopia only, and caregivers in both Ethiopia and Nepal.

**Methods:**

This was a prospective, cross-sectional study with a mixed methods design. CHWs and FLHFWs were trained to use both devices and provided with refresher training on all WHO requirements to assess fast breathing. Immediately after training, CHWs were observed using ARIDA on two children. Routine pneumonia case management consultations for children aged 5 years and younger and the device used for these consultations between the first and second consultations were recorded by CHWs in their patient log books. CHWs were observed a second time after 2 months. Semistructured interviews were also conducted with CHWs, FLHFWs, and caregivers. The proportion of consultations with children aged 5 years and younger where CHWs using an ARIDA and adhered to all WHO requirements to assess fast breathing and device manufacturer instructions for use after 2 months will be calculated. Qualitative data from semistructured interviews will be analyzed using a thematic framework approach.

**Results:**

The ARIDA project was funded in November 2015, and data collection was conducted between April and December 2018. Data analysis is currently under way and the first results are expected to be submitted for publication in 2020.

**Conclusions:**

This is the first time the usability and acceptability of automated RR counters in low-resource settings have been evaluated. Outcomes will be relevant for policy makers and are important for future research of this new class of diagnostic aids for the management of children with suspected pneumonia.

**International Registered Report Identifier (IRRID):**

DERR1-10.2196/14405

## Introduction

Acute respiratory infections (ARIs), primarily pneumonia, are the leading infectious causes of death among children aged 5 years and younger globally, accounting for an estimated 900,000 pneumonia-related deaths in 2015 [1]. Deaths from pneumonia in children result mostly from delayed presentation to appropriate health care providers and inappropriate treatment [2].

Classification of fast breathing by community health workers (CHWs) and first-level health facility workers (FLHFWs; collectively known as front-line health workers) is based on manually counting the number of breaths in 60 seconds in children aged 5 years and younger with cough and/or difficulty breathing to assess whether the respiratory rate (RR) is high enough for a particular age to prescribe antibiotics and treat suspected pneumonia, as defined by the World Health Organization (WHO) integrated management of childhood illness (IMCI) guidelines [3] for FLHFWs and their integrated community case management (iCCM) guidelines [4] for CHWs. IMCI was developed by WHO in 1995 and is an integrated approach to child health for FLHFWs that focuses on the well-being of the child aiming to reduce death, illness, and disability and promote improved growth and development among children aged 5 years and younger. iCCM is an approach recommended by WHO, United Nations Children’s Fund (UNICEF), and partners where CHWs are trained to identify and treat symptoms of pneumonia, malaria, and diarrhea in children aged 5 years and younger, as well as to detect and refer malnutrition and severely ill children to the nearest health facility. In practice, front-line health workers admit that counting the RR can be difficult as children breath irregularly and faster than adults, the child may not be calm and still for a full minute, and it is difficult to define what is and is not a breath [5]. Misclassification of the observed rate remains high [6,7], often leading to inappropriate treatment [8].

UNICEF’s acute respiratory infection diagnostic aid (ARIDA) project was initiated as a response to the call for better pneumonia diagnostic aids [9,10] and aims to identify and assess automated RR counting aids for classifying fast-breathing pneumonia for use by front-line health workers in resource-limited community settings and health facilities. The ARIDA project team conducted these field studies to test RR diagnostic aids that meet a target product profile developed by UNICEF and shared with industry, academia, and partners to encourage and guide development of new automated RR counting aids [11]. The ARIDA technical specification listed in UNICEF’s request for proposals [12] outlines that any ARIDA device must automatically detect and display the RR to aid in the classification of fast breathing in children aged 5 years and younger and include a visual indicator for notification of above or below the age-specific fast-breathing thresholds as defined by the WHO IMCI/iCCM guidelines [3,4].

**Figure 1:**
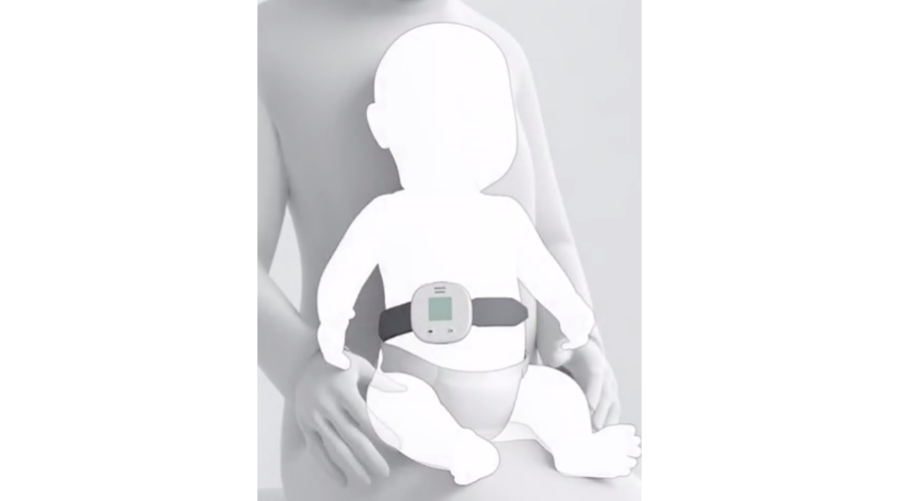
Illustration of the Children’s Automated Respiration Monitor (Koninklijke Philips NV) positioned on a child.

Two devices were newly developed that met the requirements of the UNICEF target product profile and were therefore selected for these studies. The Children’s Automated Respiration Monitor (ChARM; Koninklijke Philips NV) uses an accelerometer-based system to measure the RR in children aged 0 to 59 months and automatically classifies the breathing rate according to the iCCM/IMCI guidelines [3,4]. The device is intended to be used by front-line health workers in low-resource settings. It is strapped around the belly of the child using an elastic belt (Figure 1) and costs approximately US $50.

**Figure 2:**
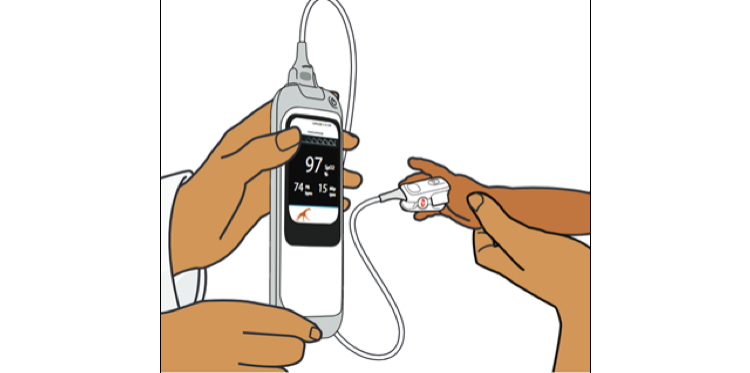
Illustration of the Rad-G pulse oximeter (Masimo) attached to a child.

The Rad-G pulse oximeter (Masimo; Figure 2) uses differential light absorbance technology to measure oxygen saturation (SpO_2_), respiration rate from plethysmograph, pulse rate, and perfusion index in children aged 0 to 59 months and classifies the breathing rate and oxygen saturation of the child according to IMCI/iCCM guidelines [3,4]. For this study, a revised iCCM algorithm to include oxygen saturation as well as RR measurements was used by the CHWs in the sick child consultations. The device has one universal probe or sensor suitable for all ages of children that is placed on the child’s finger or toe (Figure 2) and costs approximately US $250. These studies were the first time these devices were field tested for usability and acceptability.

To gather evidence around the usability of ARIDA devices to CHWs, the ChARM and Rad-G devices were evaluated in the community in Ethiopia and Nepal. Acceptability of both devices to CHWs, FLHFWs (Ethiopia only), and caregivers was evaluated.

**Figure 3:**
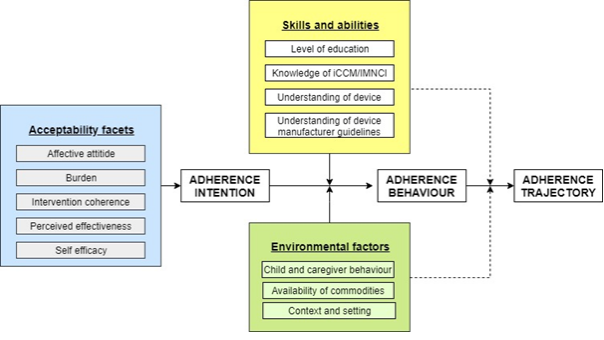
Conceptual framework of front-line health workers’ adherence to integrated community case management/integrated management of childhood illness/community-based integrated management of neonatal and childhood illness guidelines, adapted from Adams [15].

The framework in Figure 3 outlines the factors that might affect a front-line health workers’ adherence to all WHO requirements to assess fast breathing and device manufacturer instructions for use, also known as implementation fidelity [13], when using ARIDA (current and future behavior), and how they are related. A front-line health workers’ intention to adhere to guidelines is affected by 5 facets of acceptability [14]: affective attitude, burden, intervention coherence, perceived effectiveness, and self-efficacy. These acceptability facets, combined with the health workers’ skills and abilities (level of education, knowledge of relevant guidelines, understanding of how to use the device, and the device manufacturer guidelines) and other constraints (child behavior, caregiver behavior, context, and setting) will affect their adherence to guideline behavior and adherence trajectory over time.

Conceptual framework of front-line health workers’ adherence to integrated community case management/integrated management of childhood illness/community-based integrated management of neonatal and childhood illness guidelines, adapted from Adams [15].

The study aim was to understand the usability of two new automated RR diagnostic aids for CHWs and their acceptability to CHWs and caregivers in Ethiopia and Nepal (and FLHFWs in Ethiopia only) including facilitators and barriers to use.

The study objectives were as follows:

- Determine if CHWs in Ethiopia and Nepal adhere to all WHO requirements to assess fast breathing and device manufacturer instructions for use to assess and classify children aged 5 years and younger with cough and/or difficult breathing using an ARIDA
- Document the user experience of the ARIDA in a sick child consultation
- Explore the acceptability of the ARIDA to CHWs and caregivers of the sick children being assessed in Ethiopia and Nepal (and FLHFWs in Ethiopia only)

## Methods

### Study Design

**Figure 4:**
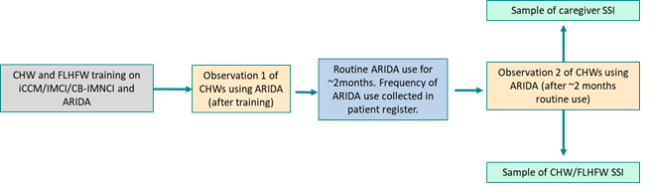
Acute respiratory infection diagnostic aid acceptability study flow diagram.

The study was a prospective, cross-sectional study with a mixed methods design that included quantitative and qualitative data collected from study participants. Prior to starting the first quantitative data collection, a training of trainers and research teams and a cascade training for CHWs/FLHFWs were conducted (Figure 4).

### Study Sites

The study was conducted in community settings and first-level health facilities in Shebedino, Dale, and Boricha districts in the Southern Nations, Nationalities, and Peoples’ Region (SNNPR), Ethiopia, and around the town of Jumla, in the Karnali region, Nepal. These two settings were selected because of the high burden of childhood pneumonia, having a mixture of rural and periurban populations, sufficient numbers of CHWs and FLHFWs with experience and availability, political stability, and availability of oxygen at district hospitals in case the children needed to be referred (Table 1).

**Table 1:** Details of study sites selected from the acute respiratory infection diagnostic aid usability and acceptability studies.

Variable	Ethiopia	Nepal
Pneumonia deaths (percentage of total deaths in children aged 5 years and younger) [16]	18%	15%
Name for CHW^a^	HEW^b^	FCHV^c^
Length of initial CHW training program provided	1 year	18 days
Literacy level	High	Extremely low
Standard practice pneumonia diagnosis tool being used	Wristwatch/ARI^d^ timer	ARI timer
WHO^e^ case management algorithms used in country	HEW: iCCM^f^; FLHFW^g^: IMCI^h^	FCHV: CB-IMNCI^i^

^a^CHW: community health worker.

^b^HEW: health extension worker.

^c^FCHV: female community health volunteer.

^d^ARI: acute respiratory infection.

^e^WHO: World Health Organization.

^f^iCCM: integrated community case management.

^g^FLHFW: first-level health facility worker.

^h^IMCI: integrated management of childhood illness.

^i^CB-IMNCI: community-based integrated management of neonatal and childhood illness.

These districts also had logistical and operational feasibility for data collection and quality assurance (QA). In Ethiopia, Malaria Consortium (implementing partner) had a strong relationship with the SNNPR regional health bureau and public health officials in Shebedino, Dale, and Boricha districts because of previous pneumonia diagnostic research and a strong presence in the UNICEF field office. In Nepal, the Karnali region was a focus area for the UNICEF country office.

### Study Populations

The studies, in Nepal and Ethiopia, included 528 children aged 5 years and younger with cough and/or difficult breathing presenting to CHWs for consultations. Also, 132 CHWs in Nepal and Ethiopia performing iCCM/community-based integrated management of neonatal and childhood illness (CB-IMNCI) at the community level participated in both the quantitative and qualitative elements of the study. In Ethiopia, the government has deployed more than 42,000 female CHWs, or health extension workers (HEWs), providing preventive, promotive, and curative health services to the community. There are typically two HEWs assigned to a health post in a subdistrict with a population of 3000 to 5000. The HEWs are supervised by health centers that oversee approximately 5 health posts each. In Nepal, CHWs are called female community health volunteers (FCHVs) [17].

The iCCM algorithm has been adapted for individual country settings, and in Nepal FCHVs are trained on a version called CB-IMNCI [18], while in Ethiopia HEWs are trained on iCCM [19]. In the CB-IMNCI in Nepal, the primary role of FCHVs is as health promoters/educators, and this includes dispensing essential commodities (eg, distribution of iron, zinc, oral rehydration solution, chlorhexidine). As per the WHO guideline, in the CB-IMNCI program in Nepal, amoxicillin is the first-line drug of choice for the treatment of neonatal sepsis and pneumonia, but more recently a national policy change has meant that this is not provided by FCHVs, who instead refer the patient to the health center to receive the treatment there. In Ethiopia, HEWs are trained for 1 year on an extended iCCM algorithm and can provide treatment of amoxicillin as required [19].

Furthermore, the CB-IMNCI program has included various social and behavioral change community level activities including demand generation activities for newborn and child health services, primarily undertaken by FCHVs. In Ethiopia, 20 FLHFWs performing IMCI at the health center level were trained to use an ARIDA and participated in semistructured interviews but were not assessed for adherence to guidelines as it was felt adequate to test usability on the lowest level of health workers. For each study, 20 caregivers of children aged 5 years and younger were also recruited to participate in the qualitative semistructured interviews.

### Sample Size

The study was powered for the primary outcome: the proportion of consultations of children aged 5 years and younger where CHWs using an ARIDA adhered to all WHO requirements to assess fast breathing and device manufacturer instructions for use after 2 months of routine use. The study used the sample size formula for a prevalence study with a specified level of confidence and precision: n=*Z*^2^*P(1–*P*)/*e*^2^, where *Z*=value from standard normal distribution corresponding to desired confidence level (*Z*=1.96 for 95% confidence interval), *P* is the expected true proportion, and *e* is the desired precision, thereby allowing a device-specific sample size to be calculated.

Assuming that the proportion of RR assessments completed correctly with an ARIDA by a CHW is 75%, and of these 95% classify the child correctly, the estimated prevalence of the primary outcome is 71%. Based on a prevalence of 71%, a sample of n=141 sick child assessments was required to estimate the true value (primary outcome) with a precision of 7.5% and 95% confidence. Applying the design effect of 1.7 and a 10% increase in the sample to account for possible clustering due to the first and second observations (posttraining and postroutine practice) and loss to follow-up of CHWs between training, respectively, a sample size of n=264 child assessments per evaluation was required (141*1.7*1.1). Thus, 132 CHWs were observed completing 2 sick children consultations twice, one directly after being trained and one subsequently after having used the device for 2 months in routine practice. This totals 528 sick child consultations for the ChARM and Rad-G usability and acceptability studies in Nepal and Ethiopia.

Semistructured interviews were conducted with a subsample of HEWs immediately after their final observation. HEWs were purposefully selected with a range of years’ experience; caregivers of children who were assessed by this subsample of HEWs were also interviewed. A convenience sample of FLHFWs available on the day we visited health facilities was used.

### Selection Criteria

Inclusion criteria for the acceptability study were any child aged 0 to 59 months with parent or guardian consent. For those aged 2 to 59 months, the child also needed to have had cough and/or difficulty breathing. Exclusion criteria for children in all elements of the study were those with general danger signs of convulsions or no movement for children aged 0 to 59 months and lethargy or unconsciousness for children aged 2 to 59 months [3], those with a parent or guardian aged younger than 16 years, those not having parent or guardian consent, or those having any device manufacturer safety exclusion criteria. For ChARM, these were preterm babies (born before 37 weeks of gestation), children wearing a supportive device at area of chest/belly, or children whose skin was not intact on their chest/belly [20]; for Rad-G, these were children whose skin was not intact at the application site (finger or toe).

### Outcomes

The primary outcome was the proportion of consultations on children aged 5 years and younger where CHWs using an ARIDA adhered to all WHO requirements to assess fast breathing and device manufacturer instructions for use after 2 months of routine use. Secondary outcomes included the difference in proportion of consultations on children aged 5 years and younger where CHWs using an ARIDA adhered to WHO requirements to assess fast breathing and device manufacturer instructions for use immediately after training and after 2 months of routine use; number of errors made during the consultation of the sick child (assessment, classification, treatment, referral) immediately after training and after 2 months of using an ARIDA routinely; mean time taken to complete the sick child consultation; number of unsuccessful attempts using an ARIDA; number of times no ARIDA reading could be obtained in up to 3 attempts; number of children assessed for respiratory signs and symptoms by CHWs with an ARIDA during routine practice; and for Rad-G, number of children assessed for respiratory signs and symptoms by CHWs with standard practice in the same period of the previous year. Qualitative outcomes focused on acceptability were derived from the 7 facets of acceptability as presented in the work by Sekhon et al [21].

### Training

A 2-day joint training was held for the country-level research teams of project manager, data manager, and 7 teams each consisting of 2 research assistants and the trainers of CHWs/FLHFWs. This training was led by the Malaria Consortium capacity building specialist and supported by the relevant Ministry of Health regional health authorities, UNICEF country office, and the global ARIDA project team. The 2 days focused on introducing the team to the ARIDA study, required WHO case management and manufacturer RR instructions for use, and how to use the ARIDA devices. The research teams and trainers then separately attended a third day of training which was more specific to their role in the study. The 7 research teams focused on the study procedures including the informed consent process, how to use the CommCare data collection application (version 2.38.1, Dimagi Inc), data collection and management procedures, and QA procedures. Before the second observation, there was a 1-day training on conducting the qualitative semistructured exit interviews with caregivers, HEWs, and FLHFWs for all research teams led by the Malaria Consortium project team. In their training-of-trainers session, the 7 trainers focused on developing the modules for the CHW/FLHFW training and adult learning techniques. The 132 CHWs in each study were trained in 6 cohorts of approximately 22 CHWs each. In Ethiopia, a seventh FLHFW training for 20 FLHFWs was also done. Each cohort was trained over 2 days by one trainer supported by a member of the Malaria Consortium project team, implementing partner project team, and local research teams, as required. The training consisted of the following modules: introduction to training, ARIDA study overview, assessment of fast breathing with an ARIDA, field evaluation data forms, case management of pneumonia, and ethical considerations and subject safety. CHWs/FLHFWs were guided through the information giving and consent process. For each study, a comprehensive job aid was developed and provided to each health worker. The job aid contained all the information to conduct the required WHO case management and device manufacturer instructions for use, along with further information on pneumonia prevention (Multimedia Appendix 1). The training included a half-day practice session that allowed each health worker to practice using the device on a range of different aged children. All CHWs had to pass a competency-based assessment at the end of the training in order to be included in the study. This involved a 12-question assessment including a practical exam on device use, with a pass mark of 75%. One research team and trainer participated in a 5-day master pretest of all procedural activities that followed the exact procedures of the study to ensure all members of the research team and trainers were conversant with the study procedures and data collection materials. The pretest piloted the training of CHWs/FLHFWs, subject screening, subject consent, quantitative evaluation, data collection with the draft data collection tools, data entry into the CommCare data collection application, and the log-book review. The research team visited up to 3 research sites with a trainer and conducted the quantitative assessment. There was a 5-day debrief, revision, and finalization of all quantitative study data collection materials after the pretest, plus translation of relevant study materials. There was a second 1-day pretest for all 7 quantitative research assistants to practice using the finalized data collection tools. Within each research team, one research assistant participated in a 1-day pretest of qualitative data collection tools before the second quantitative evaluation during the 2 months of routine data collection. Each research assistant conducted a semistructured interview with 2 CHWs/caregivers. There was a 1-day debrief, revision, and finalization of the qualitative data collection materials after the pretest, plus translation of relevant study materials.

### Study Procedures

The ChARM assessments took place between May and July 2018 in Ethiopia and between September and December 2018 in Nepal. The Rad-G assessments took place between September and December 2018 in Ethiopia. Patients were screened by the CHWs using the ARIDA job aid, children were enrolled prospectively based on eligibility, and the research assistants obtained the parent or guardian’s consent.

For each consultation, 2 research assistants independently observed the CHW conduct the sick child consultation and silently recorded their actions on tablet-based observation checklists. In some instances, the research assistants needed to capture source documentation in the form of photographs of the age group selected and the ARIDA RR reading. The research assistants also took photographs of the patient registers for later review. Once the evaluation was completed, the research assistants gave feedback to the CHWs if they had observed any incorrect actions. The CHWs’ actions for the 8 steps involved in using ChARM were recorded (Table 2).

There were also 8 steps that were observed when the Rad-G was used (Table 3).

**Table 2:** Steps of the child consultation that community health workers using the Children’s Automated Respiration Monitor were observed completing.

Consultation step	Definition	Category
Correct child position	Back fully supported, either in the arms of the caregiver (younger child), sitting on caregiver’s lap with their back against the caregiver’s front (older child), or lying on their back on a flat surface (older child)	Device manufacturer instructions for use
Correct device position	Device on the belly line in line with the nipple	Device manufacturer instructions for use
Correct belt attachment	ChARM^a^ touching skin/clothing and belt not tangled	Device manufacturer instructions for use
Correct age group	Age group selected by HEW^b^ on ChARM matches screening checklist	WHO^c^ requirements to assess fast breathing
Correct child behavior immediately before ChARM attempt	Calm: not actively crying or moving	WHO requirements to assess fast breathing
Correct child eating/breastfeeding status during successful ChARM attempt	No eating/breastfeeding	WHO requirements to assess fast breathing
Correct child behavior during successful ChARM attempt	Calm: not actively crying or moving	WHO requirements to assess fast breathing
Correct classification	According to CB-IMNCI^d^ guidelines, based on screening age group and breathing status of the child	WHO requirements to assess fast breathing

^a^ChARM: Children’s Automated Respiration Monitor.

^b^HEW: health extension worker.

^c^WHO: World Health Organization.

^d^CB-IMNCI: community-based integrated management of neonatal and childhood illness.

**Table 3:** Steps of the child consultation that community health workers using Rad-G were observed completing.

Consultation step	Definition	Source of step
Child calm before assessment	Calm: not actively crying or moving	WHO^a^ requirements to assess fast breathing
Correct mode selected	Screening mode	Device manufacturer instructions for use
Correct age group	Age group recorded by HEW^b^ on Rad-G device matches screening checklist	WHO requirements to assess fast breathing
Correct probe position	Fully inserted	Device manufacturer instructions for use
Correct probe direction	Picture on top of finger or toe	Device manufacturer instructions for use
Child not eating/feeding during assessment	No eating/breastfeeding	WHO requirements to assess fast breathing
Child calm during assessment	Calm: not actively crying or moving	WHO requirements to assess fast breathing
Correct classification	According to iCCM^c^ guidelines, based on screening age group and breathing status of the child	WHO requirements to assess fast breathing

^a^WHO: World Health Organization.

^b^HEW: health extension worker.

^c^iCCM: integrated community case management.

The total time taken to get a successful ARIDA reading was recorded using a stopwatch. Once the assessment was completed, the CHW recorded the result of the RR and informed the research assistants of the classification and treatment. If an RR count could not be obtained on the first attempt, the attempt was recorded as unsuccessful and repeated up to two more times before the CHW moved to current practice (ARI timer, phone, watch).

On completion of the assessment, the CHW explained the classification to the caregiver and gave them treatment, referral, or home care advice as appropriate. CHWs were asked to use ARIDA during the 2 months of routine use but could revert to standard practice if they needed to and were instructed to record which device they used in their patient register using colored stickers (one patient register per health post).

### Data Collection

Quantitative data was collected using an electronic data collection platform (CommCare) installed onto password-protected 7C Pro (Tecno Mobile) tablets and backed up to a protected cloud server. Unique identification codes were used to anonymize patient data and CHW data. All RR evaluation data were independently entered by each research assistant. The data manager downloaded data daily and entered it into a data checker with in-built validation checks. Trial conduct was audited internally and externally. In Ethiopia, Malaria Consortium (United Kingdom and Ethiopia) and UNICEF (Supply Division and Ethiopia Country Office) conducted weekly QA visits to the research site during data collection. In Nepal, Malaria Consortium (UK), UNICEF Country Office, and HERD International (Nepal implementing partner) also conducted QA visits to the research site during data collection. Malaria Consortium created a QA form template (Multimedia Appendix 2) which was completed by team members when shadowing the research teams. All data collected by research assistants from the CHW assessments was checked and verified by the Malaria Consortium research team daily. A 1-day refresher training was provided to the quantitative research team before the start of the second data collection. Qualitative data was audiorecorded for each semistructured interview, and all audio recordings were translated into English and transcribed by the research assistants.

The project had an 11-person advisory committee of global experts to facilitate dissemination and uptake of any findings within participating countries as well as with key partners in the global childhood pneumonia community.

### Data Analysis

Descriptive information about the CHWs will be presented including CHW participation (number trained and number completing first and second observation), sex, district, number of years qualified as a CHW, CHW education level, last integrated refresher training and last supervision (Ethiopia only), and literacy level (Nepal only). Descriptive information about the number of children enrolled, number of evaluations started, number of evaluations completed by ARIDA and standard practice, and child age and sex will also be presented.

The primary outcome will be calculated as the proportion of consultations with children aged 5 years and younger where CHWs using an ARIDA adhered to all WHO requirements to assess fast breathing and device manufacturer instructions for use after 2 months of routine use. This analysis will be disaggregated by age group, breath rate, and SpO_2_ classification (Rad-G only). Secondary outcomes including the proportion of CHWs correctly performing steps reflecting the device manufacturer instructions for use and steps that reflect all WHO requirements to assess fast breathing will also presented, as will the difference in the proportion of consultations that were completed correctly between observation 1 and observation 2. For the main outcomes, the most conservative estimates will be used (ie, if the two research assistants disagreed on how the CHW performed a step in the assessment, the one that recorded an inconsistency/error for that step was used over the one who recorded that the step was performed correctly). A sensitivity analysis using less conservative observations will also be presented. The mean time taken to complete the full assessment will be calculated for ChARM as from the time the CHW straps on the device to when the device displays an RR reading. For Rad-G, it will be from the time when the CHW turns on the Rad-G (prior to probe placement) to when the Rad-G displays an SpO_2_ and RR reading. The number of children who were assessed for signs of respiratory illness by CHWs with ARIDA or standard practice during routine care will also be presented. For the qualitative analysis, semistructured interview data will be summarized and presented for caregivers and front-line health workers separately. All qualitative data will be analyzed using MAXQDA (VERBI GmbH). Transcripts will be loaded into the software and a coding frame for the CHW/FLHFW interviews and caregiver interviews developed. Underlying themes should emerge iteratively. Each theme will be critically analyzed by the research team until the final themes are agreed upon.

### Ethical Approval and Consent to Participate

The study was approved by ethical review boards in each study country at national or regional level: in Ethiopia, from the SNNPR Health Bureau Health Research Review Committee (ref -241/20852) on May 4, 2018; in Nepal, from the Nepal Heath Research Council (Ref 2334); and in the United Kingdom, from the Liverpool School of Tropical Medicine (Ref 18-026) on July 10, 2018. Participants and health workers were recruited into the study only after written informed consent.

## Results

The ARIDA project was funded in November 2015, and data collection was conducted between April and December 2018. Data analysis is currently under way, and the first results are expected to be submitted for publication in 2020.

## Discussion

### Summary

The aim of this study is to understand usability (Can CHWs in Ethiopia and Nepal adhere to all WHO requirements to assess fast breathing and device manufacturer instructions for use to assess and classify children aged younger than 5 years with cough and/or difficult breathing using ARIDA after 2 months of routine use?) and acceptability (ie, front-line health worker and caregiver perceptions on benefits and barriers to ARIDA use). This is the first study that has been done to build the evidence base around the usability and acceptability of new automated RR diagnostic aids in low-resource settings.

### Strengths and Limitations

A potential strength of the study is that we used two research assistants to observe the CHWs’ consultations and can perform a sensitivity analysis to understand how disagreement between research assistants could affect adherence rate for different assessment stages. Another strength is that we measured adherence to the algorithm after 2 months without any refresher training, which should provide evidence on how adherence could change between training and second evaluation with no additional support.

A study limitation could be the Hawthorne effect [22] where the CHWs could change their behavior because the research assistants are observing the consultation. In the study procedure, the research team took steps to minimize this by silently observing child consultations and not interfering with the consultations. There was also the potential for response bias from the self-reported routine data collected through the patient register from the CHWs who may have had a tendency to inflate their reporting of the number of times they used the ARIDA.

### Conclusion

We hope that the results of this study will add to the evidence base for automated RR counters and support decision making around their adoption and increased use in these settings and among these types of health workers. The findings can also be used to help device manufacturers in the development and refinement of such technologies for use in low-resource settings.

## Appendix

Multimedia Appendix 1 Rad-G job aid, Ethiopia.

Multimedia Appendix 2 Quality assurance assessment form.

## Abbreviations

ARI: acute respiratory infection
ARIDA: acute respiratory infection diagnostic aid
CB-IMNCI: community-based integrated management of neonatal and childhood illness
ChARM: Children’s Automated Respiration Monitor
CHW: community health worker
FCHV: female community health volunteer
FLHFW: first-level health facility worker
HEW: health extension worker
iCCM: integrated community case management
IMCI: integrated management of childhood illness
QA: quality assurance
RR: respiratory rate
SNNPR: Southern Nations, Nationalities, and Peoples’ Region
SpO_2_: oxygen saturation
UNICEF: United Nations Children’s Fund
WHO: World Health Organization
