# Supplementary material for: Automated Respiratory Rate Counter to Assess Children for Symptoms of Pneumonia: Protocol for Cross-Sectional Usability and Acceptability Studies in Ethiopia and Nepal
Source: JMIR Res Protoc. 2020 Mar 30;9(3):e14405. doi: 10.2196/14405 (PMC7154937; doi:10.2196/14405)
Supplement: Multimedia Appendix 1 [file resprot_v9i3e14405_app1.pdf]

የራድ ጂ የስራ መርጃ

Rad-G Device - Job Aid

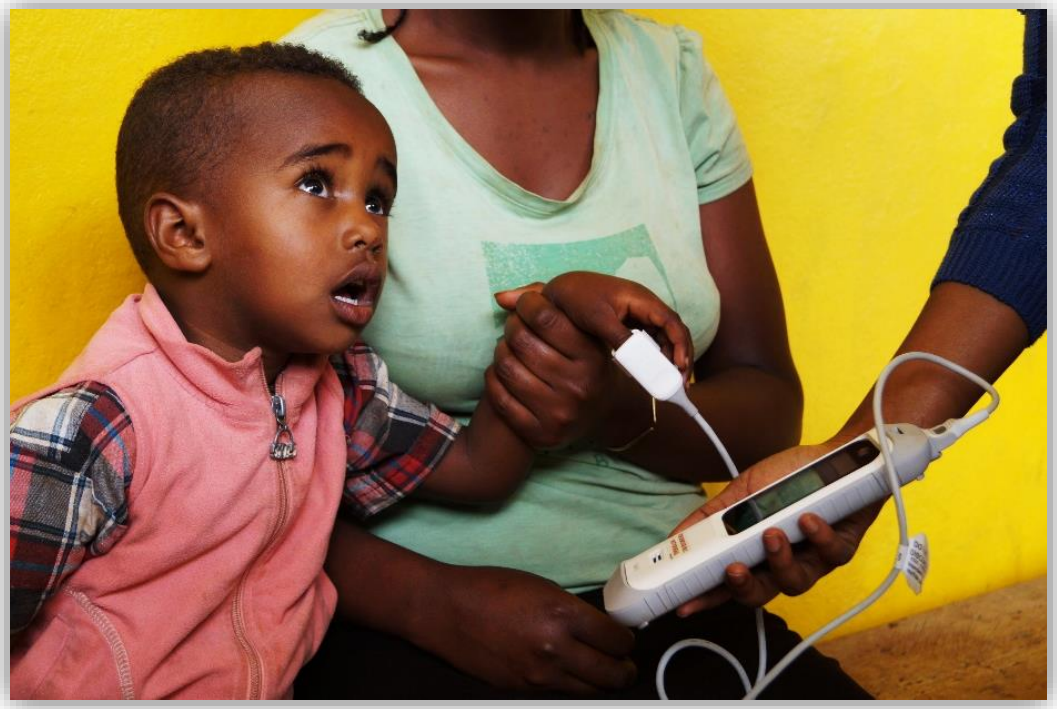

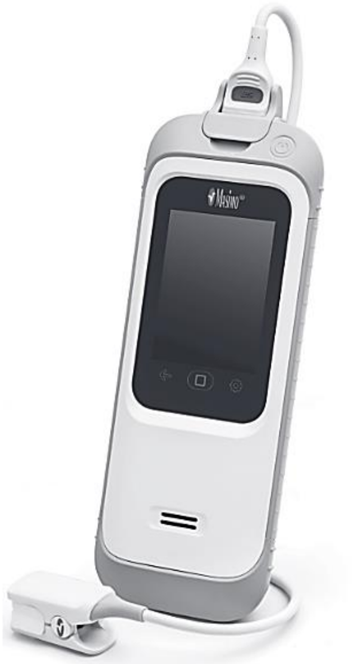

የምርመራ መሳሪያ ኢትዮጵያ

Diagnostic Device Ethiopia

"la Caixa" Banking Foundation

unicef

malaria consortium
disease control, better health

የህፃኑን ዕድሜና ክብደት ማጣራት

STEP 1: Assess the child's age and weight

የህፃኑን ዕድሜ ጠይቂ

Ask the child's age

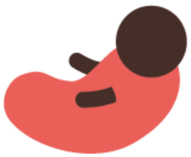

ከ0-2 ወር ጨቅላ ህፃን

Young infant 0 to <2 months

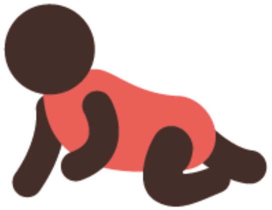

ከ2-12 ወር የሆነው ህፃን

Infant 2 to <12 months

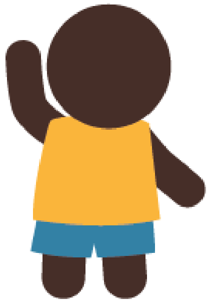

ከ1-5 ዓመት የሆነው ህፃን

Child 12 to 59 months

በየትኛዉም ዕድሜ ለሚገኙ ሕፃናት ክብደታቸዉን ይለኩ

Obtain child's weight for all ages

malaria consortium
disease control, better health

አደገኛ ምልክቶችን መመርመር 0-2 ወራት

STEP 2: Assess for signs of Very Severe Disease: 0 to < 2 months

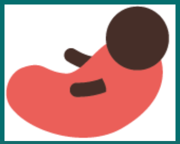

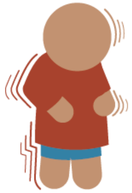

ጨቅላዉ ኮንቨልሽን ካለዉ

The infant has convulsions now

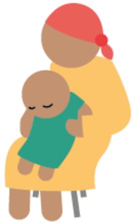

ጨቅላዉ ሲነኩት ብቻ የሚንቀሳቀስ ወይም ሲነካም የማይንቀሳቀስ

The infant is moving only when stimulated or not at all moving

→ **በጥናቱ አይካተትም ለታካሚዉ ጨቅላ የቅድመ ሪፈራል መድሃኒት በመስጠት በአፋጣኝ ወደ ሆስፒታል ሪፈር አድርጊ**

Exclude. Give infant pre-referral dose and refer immediately to hospital

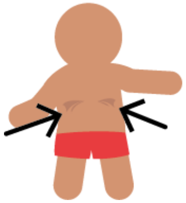

ጨቅላዉ ከባድ የደረት መሰርጎድ አለው

The infant has chest indrawing

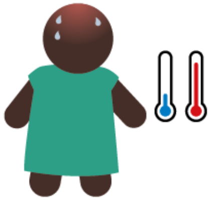

ጨቅላዉ የሰውነት ሙቀት መጠን ይለኩ:: የሙቀት መጠኑ ≥37.5ከሆነ ትኩሳት ነው:: የሙቀት መጠኑ <35.5 c ከሆነ ዝቅተኛ የሙቀት መጠን ነው::

Obtain axillary temperature.
The infant has fever ≥37.5 c
or low body temperature <35.5 c

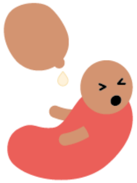

ጨቅላው በደንብ መጥባት አቅቶታል

The infant has stopped feeding well

→ **ወደ ተግባር 3 ይቀጥሉ:**

Proceed to Step 3: Assess RR and SpO2 with Rad-G

malaria consortium
disease control, better health

አደገኛ ምልክቶችን መመርመር 2-59 ወራት

STEP 2: Assess for Danger Signs: 2 to 59 months

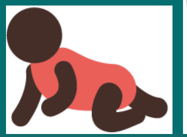

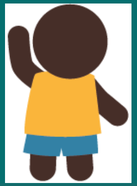

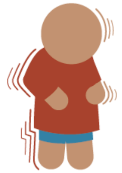

ህፃኑ አሁን ኮንቨልሽን አለዉ?

The child is convulsing now

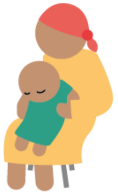

ህፃኑ የፈዘዘ ወይም ራሱን የሳተ ነዉ?

The child is lethargic or unconscious

→ **በጥናቱ አይካተትም ለታካሚዉ ሕፃን የቅድመ ሪፈራል መድሃኒት በመስጠት በአፋጣኝ ወደ ሆስፒታል ሪፈር አድርጊ**

Exclude. Give pre-referral dose and refer immediately to hospital.

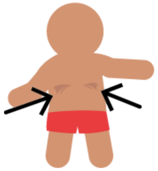

የደረት መሰርጎድ / ስትራይደር አለው

The child has chest indrawing or stridor

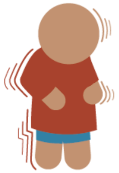

ህፃኑ በዝህኛዉ ህመሙ ጊዜ ኮንቨልሽን ነበረዉ?

The child has had convulsions

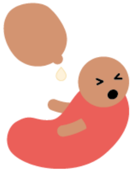

ህፃኑ መጠጣት / ጡት መጥባት አቅቶታል?

The child is unable to drink or breastfeed

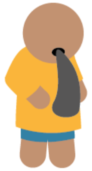

ህፃኑ የበላዉን እና የጠጣዉን በሙሉ እና በወሰደ ቁጥር ያስመልሰዋል ?

The child vomits everything

→ **ወደ ተግባር 3 ይቀጥሉ፡**

Proceed to Step 3: Assess RR and SpO2 with Rad-G

malaria consortium
disease control, better health

የህፃኑን የሕህመም ሁኔታ መመርመር

STEP 3: Assess the child's history of Illness

ዕድሜአቸዉ ከ2 እስከ 59 ወራት ለሆኑ ሕፃናት:-ሣል እና/ወይንም የአተነፋፈስ ችግር ካለበትና ምን ያህል ግዜ እንደቆየበት ይጠይቁ

ስትራይደር እንዳለ ተመለከቺ አዳምጪ

Child aged 2 to 59 months:
Ask about cough and difficulty breathing and for how long?
Look and listen for stridor

ሳል እና/ወይንም የአተነፋፈስ ችግር የለበትም

No history of cough and/or difficulty breathing

በጥነቱ አይካተትም በአይሲሲኤም መሰራት ምርመራውን ቀጥይ

Exclude from Rad-G and continue with child assessment

ህፃኑ ከ2 ሳምንት በታች በሆነ ጊዜ ውስጥ ሳል እና/ወይም የመተንፈስ ችግር ካለበት ወደ ተግባር 4 ቀጥይ

If the child has history of cough and/or difficulty breathing of less than 2 weeks continue to Step 4

malaria consortium
disease control, better health

በራድ ጂ መሳሪያ ኦከስጂን መጠንና ትንፋሽ መጠንን መለካት

STEP 4: Assess RR and SpO2 using the Rad-G Device

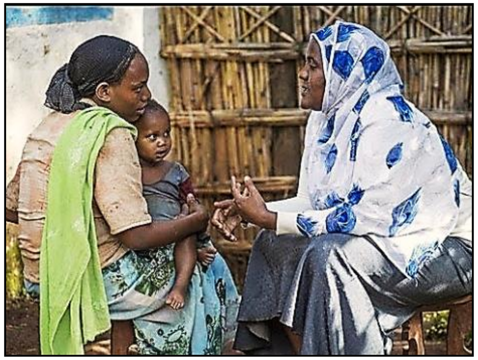

ለሕፃኑ ወላጅ/አሳዳጊ የራድ-ጂ መሣሪያን በመጠቀም ምርመራ እንደምታካሂጂ በሚገባ አብራሪላቸዉ

Explain to the caregiver that you will use the Rad-G device

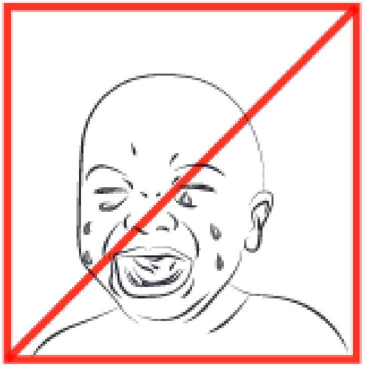

ህፃኑ እስኪረጋጋ ጠብቁ

Wait for the child to be calm

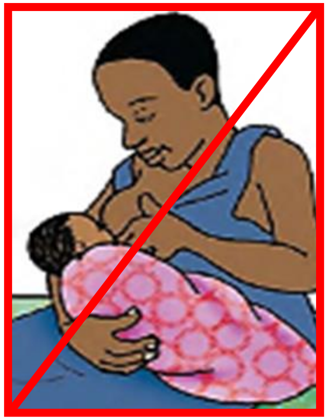

ህፃኑ ጡት መጥባት የለበትም

The child should not be feeding

malaria consortium
disease control, better health

በራድ ጂ መሳሪያ አከሰጂን መጠንና ትንፋሽ መጠንን መለካት

STEP 4: Assess RR and SpO2 using the Rad-G Device

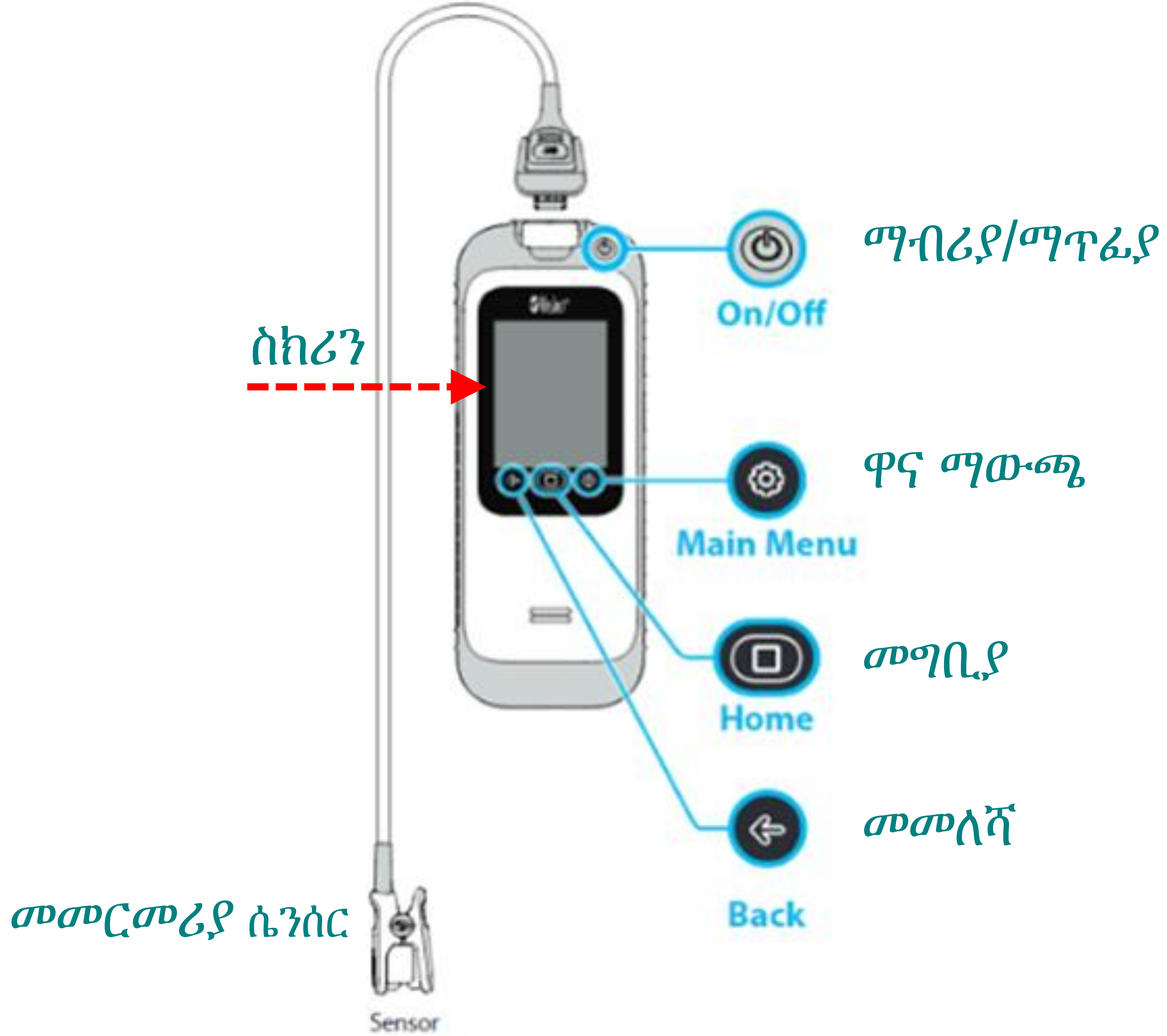

malaria consortium
disease control, better health

በራድ ጂ መሳሪያ አከስጂን መጠንና ትንፋሽ መጠንን መለካት

STEP 4: Assess RR and SpO2 using the Rad-G Device

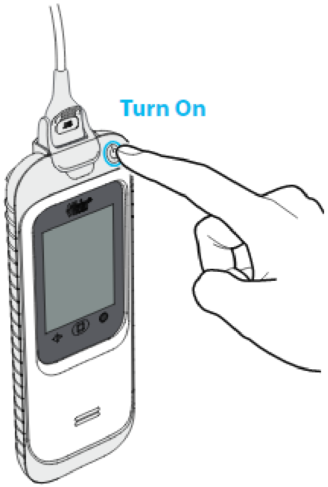

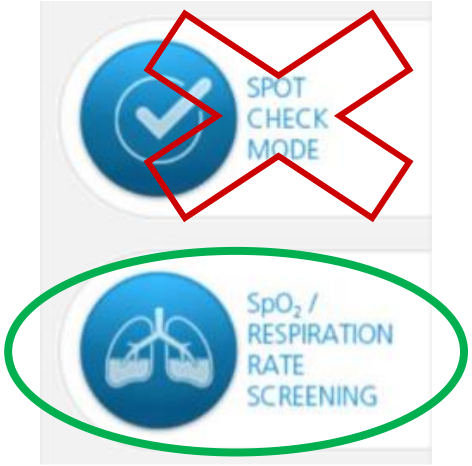

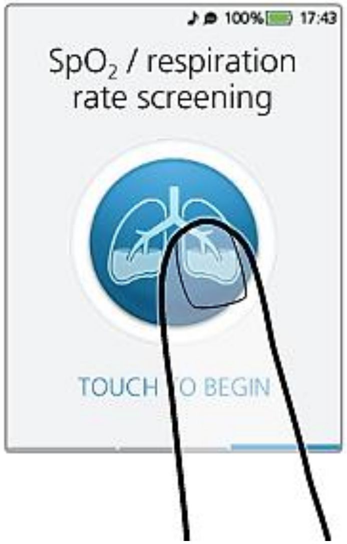

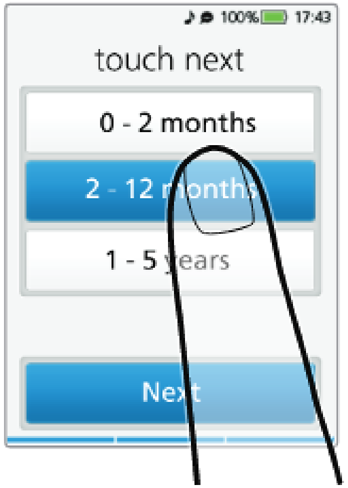

ማብሪያ ማጥፊያውን በመጫን መሳሪያውን አብሪ

Turn on the device

የሳንባ ምስል ያለበትን የምርመራ አማራጭን ምረጪ

Select the screening mode

እስክሪኑ ላይ የሳንባ ምስል ያለበትን በመጫን አስጀምሪ

Touch the lung icon on the screen to begin

የህፃኑን ዕድሜ ከመረጥሽ በኋላ Next የሚለውን ንኪ

Select age group and press ‘Next’

malaria consortium
disease control, better health

በራድ ጂ መሳሪያ አከሰጂን መጠንና ትንፋሽ መጠንን መለካት

STEP 4: Assess RR and SpO2 using the Rad-G Device

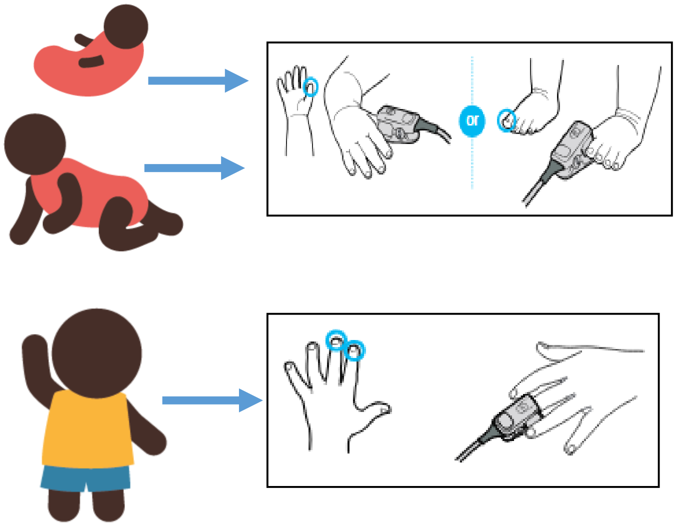

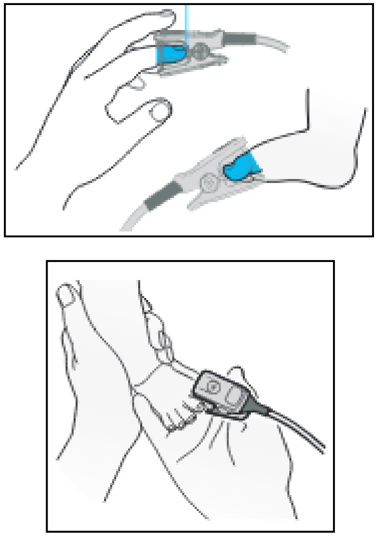

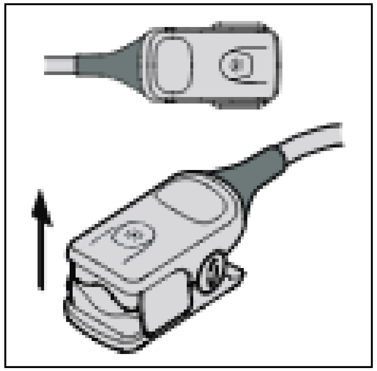

ሴንሰሩ ንፁህ መሆኑን አረጋግጪ
የህፃኑን የእጅ ጣት ወይም የእግር
አውራ ጣት በማፅዳት
ከመመርመሪያው ሴንሰር ጋር አያይዢ

Check the sensor to make sure it is clean. Select the child's toe, finger or thumb for best fit and clean with alcohol swab before applying probe

የህፃኑን ጣት በመመርመሪያው
ሴንሰሩ ላይ ሙሉ በሙሉ
እንዲገባ በማድረግ
ህፃኑ እንዳይንቀሳቀስ አድርጊ

Fully insert the probe and ensure there is no movement

የጣት ምልክት ያለበት
የመመርመሪያው
ሴንሰር ገፅ ከላይ በኩል
መሆን ይኖርበታል

The probe needs to have the picture facing up

malaria consortium
disease control, better health

በራድ ጂ መሳሪያ አከሰጂን መጠንና ትንፋሽ መጠንን መለካት

STEP 4: Assess RR and SpO2 using the Rad-G Device

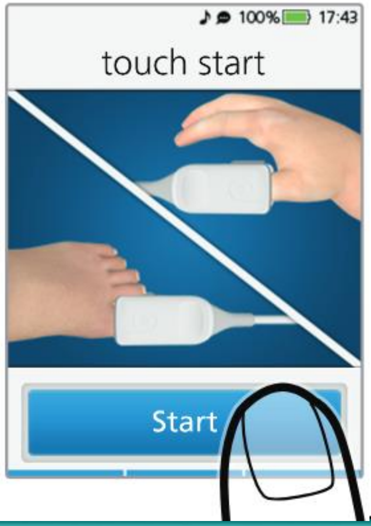

Start የሚለውን በመንካት አስጀምሪ

Press 'Start'

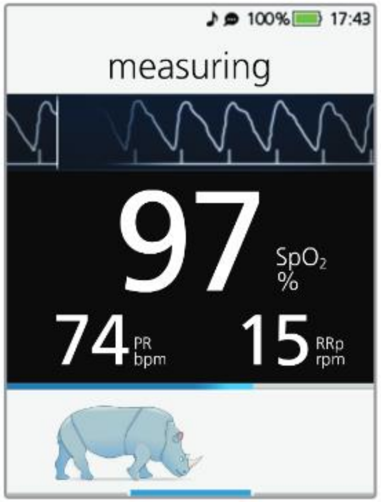

ውጤት እስከሚታይ ጠብቂ

Wait for the results

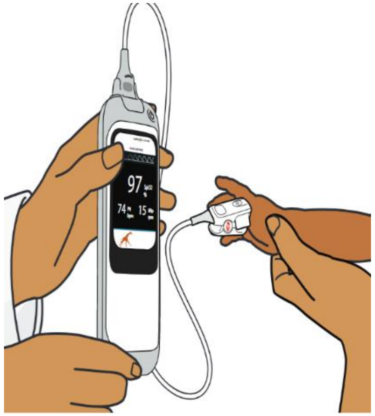

ውጤት እስከሚታይ ስክሪኑን በማሳየት ህፃኑን ማጫወት ትቺያለሽ

Show the screen to the child for entertainment

በራድ ጂ መሳሪያ ውጤት ለማግኘት እስከ 3 ሙከራ መደረግ ይኖርበታል፡፡ ከ3 ሙከራ በኋላ በተለመደው የትንፋሽ መቁጠሪያ መጠቀም ይቻላል፡፡

If you are unsuccessful with Rad-G, try up to 3 times before using your normal practice

malaria consortium
disease control, better health

የራድ ጂ ውጤትን መተንተን

STEP 5: Interpret the Rad-G results

የስክሪን ቀለም<br>Screen Colour	የኦክስጂን መጠን<br>SpO2 Results	ትንፋሽ በደቂቃ<br>RR results	ውጤቱ ምን ማለት ነው<br>Interpretation
አረንጓዴ<br>Green	≥90%	ከ 0 እስከ 2 ወራት 59 ትንፋሽ በደቂቃ ወይንም በታች<br>ከ 2 እስከ 12 ወራት 49 ትንፋሽ በደቂቃ ወይንም በታች<br>ከ 12 እስከ 59 ወራት 39 ትንፋሽ በደቂቃ ወይንም በታች<br>0 to <2 months 59 breaths or less<br>2 to <12 months 49 breaths or less<br>12 to 59 months 39 breaths or less	ኔጌቲቭ<br>Negative
ቀይ<br>Red	<90%	ከ 0 እስከ 2 ወራት 60 ትንፋሽ በደቂቃ ወይንም በላይ<br>ከ 2 እስከ 12 ወራት 50 ትንፋሽ በደቂቃ ወይንም በላይ<br>ከ 12 እስከ 59 ወራት 40 ትንፋሽ በደቂቃ ወይንም በላይ<br>0 to <2 months 60 breaths or more<br>2 to <12 months 50 breaths or more<br>12 to 59 months 40 breaths or more	ፖዘቲቭ<br>Positive
ቀይ<br>Red	≥90%	ከ 0 እስከ 2 ወራት 60 ትንፋሽ በደቂቃ ወይንም በላይ<br>ከ 2 እስከ 12 ወራት 50 ትንፋሽ በደቂቃ ወይንም በላይ<br>ከ 12 እስከ 59 ወራት 40 ትንፋሽ በደቂቃ ወይንም በላይ<br>0 to <2 months 60 breaths or more<br>2 to <12 months 50 breaths or more<br>12 to 59 months 40 breaths or more	ፖዘቲቭ<br>Positive
ቀይ<br>Red	<90%	ከ 0 እስከ 2 ወራት 59 ትንፋሽ በደቂቃ ወይንም በታች<br>ከ 2 እስከ 12 ወራት 49 ትንፋሽ በደቂቃ ወይንም በታች<br>ከ 12 እስከ 59 ወራት 39 ትንፋሽ በደቂቃ ወይንም በታች<br>0 to 2 months 59 breaths or less<br>2 to <12 months 49 breaths or less<br>12 to 59 months 39 breaths or less	ፖዘቲቭ<br>Positive

malaria consortium
disease control, better health

ከ0 እስከ 2 ወራት በከባድ ህመም ምልክቶች፣ በኦክስጅን እና አተነፋፈስ መጠን መሰረት መሰየም

STEP 6: Classify infant 0 to <2 months based on SpO2, RR results, and signs of very severe disease

የከባድ በሽታ ምልክቶች<br>Signs of very severe disease 0 to <2 months	የትንፋሽ መጠን<br>RR	የኦክስጅን መጠን<br>SpO2	ስያሜ<br>Classification	ህክምና<br>Treatment
• ጡት በደንብ መጥባት ያቃተው ወይም<br>• ትኩሳት ≥37.5 ዲ.ሴ ካለው ወይም የሚቀዘቅዝ< 35 ዲ.ሴ ከሆነ ወይም<br>• ከባድ የደረት መስርጎድ ያለው<br>• Chest indrawing, OR<br>• Fever (≥37.5 C or feels hot), OR<br>• Low body temperature (35.5 C or feels cold), OR<br>• Not feeding well	ፈጣን አተነፋፈስ (≥ 60 ትንፋሽ በደቂቃ) ወይንም<br>ፈጣን ያልሆነ አተነፋፈስ (≤ 59 ትንፋሽ በደቂቃ)<br>Fast breathing (≥60 breaths)<br>OR<br>Normal breathing (≤59 breaths)	የኦክስጅን መጠን ዝቅተኛ አይደለም (>90%)<br>SP >90%	በጣም ከባድ በሽታ<br>VERY SEVERE DISEASE	የቅድመ ሪፈራል መድሀኒት መስጠትና በአፋጣኝ ኦክስጂን ወዳለበት ሆስፒታል ሪፈር ማድረግ<br>Give pre-referral dose if appropriate and REFER URGENTLY to hospital with oxygen therapy
		ዝቅተኛ የኦክስጅን መጠን (≤90%)<br>SP ≤90%	በጣም ከባድ በሽታ እና ዝቅተኛ የኦክስጅን መጠን<br>VERY SEVERE DISEASE with HYPOXIA	
የከባድ በሽታ ምልክቶች የሌለው<br>No other signs of very severe disease	ፈጣን አተነፋፈስ (≥ 60 ትንፋሽ በደቂቃ)<br>Fast breathing (≥60 breaths)	የኦክስጅን መጠን ዝቅተኛ አይደለም(>90%)<br>SP >90%	በጣም ከባድ በሽታ<br>VERY SEVERE DISEASE	
		ዝቅተኛ የኦክስጅን መጠን (≤90%)<br>SP ≤90%	በጣም ከባድ በሽታ እና ዝቅተኛ የኦክስጅን መጠን<br>VERY SEVERE DISEASE with HYPOXIA	
የከባድ በሽታ ምልክቶች የሌለው<br>No signs of very severe disease	ፈጣን ያልሆነ አተነፋፈስ (≥ 59 ትንፋሽ በደቂቃ)<br>Normal breathing (≤59 breaths)	ዝቅተኛ የኦክስጅን መጠን<br>SP ≤90%	ዝቅተኛ የኦክስጅን መጠን<br>(የከባድ በሽታ ምልክቶች እና ፈጣን አተነፋፈስ የሌለው)<br>HYPOXIA with no signs of severe disease or fast breathing	በአፋጣኝ ኦክስጂን ወዳለበት ሆስፒታል ሪፈር ማድረግ<br>Refer to hospital for oxygen therapy
የከባድ በሽታ ምልክቶች የሌለው<br>No signs of very severe disease	ፈጣን ያልሆነ አተነፋፈስ (≥ 59 ትንፋሽ በደቂቃ)<br>Normal breathing (≤59 breaths)	የኦክስጅን መጠን ዝቅተኛ አይደለም (>90%)<br>SP >90%	ከባድ በሽታ የሌለው<br>SEVERE DISEASE UNLIKELY	የቤት ውስጥ ክብካቤ እነድታደርግ ለእናትየው ምክር ስጪ<br>Advise mother to give homecare

2 to 12 ወራት በከባድ ህመም ምልክቶች፣ በኦክስጅን እና አተነፋፈስ መጠን መሰረት መሰየም

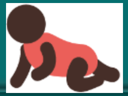

STEP 6: Classify child 2-12 months based on SpO2, RR results, and danger signs

የከባድ በሽታ ምልክቶች<br>Signs of illness 2 to 12 months	የትንፋሽ መጠን<br>RR	የኦክስጅን መጠን<br>SpO2	ስያሜ<br>Classification	ህክምና<br>Treatment
• ሳል ወይም የመተንፈስ ችግር ያለው እና<br>• በተረጋጋ ዕፃን ስትራይደር ያለው ወይም<br>• የአደገኛ በሽታ ምልከቶች ያለው ወይም<br>• የደረት መሰርጎድ ያለው<br>• History of cough or difficulty breathing, AND<br>• Stridor in calm child, OR<br>• Any general danger sign OR<br>• chest indrawing	ፈጣን አተነፋፈስ (≥ 50 ትንፋሽ በደቂቃ)<br>ወይንም<br>ፈጣን ያልሆነ አተነፋፈስ (≤49 ትንፋሽ በደቂቃ)<br>Fast breathing (≥50 breaths)<br>OR<br>Normal breathing (≤49 breaths)	የኦክስጅን መጠን ዝቅተኛ አይደለም<br>(>90%)<br>SP >90%	ከባድ የሳንባ ምች ወይም በጣም ከባድ በሽታ<br>SEVERE PNEUMONIA or VERY SEVERE DISEASE	የቅድመ ሪፈራል መድሀኒት መስጠትና በአፋጣኝ አክስጂን ወዳለበት ሆስፒታል ሪፈር ማድረግ<br>Give first dose of an appropriate antibiotic and REFER URGENTLY to hospital with oxygen therapy
		ዝቅተኛ የኦክስጅን መጠን (≤90%)<br>SP ≤90%	ከባድ የሳንባ ምች ወይም በጣም ከባድ በሽታ እና ዝቅተኛ የኦክስጅን መጠን<br>SEVERE PNEUMONIA or VERY SEVERE DISEASE with HYPOXIA	
• ሳል ወይም የመተንፈስ ችግር ያለው እና<br>• ስትራይደር ያሌለው ወይም<br>• የአደገኛ በሽታ ምልከቶች የሌለው ወይም<br>• የደረት መሰርጎድ የሌለው<br>History of cough or difficulty breathing<br>AND NO general danger signs, NO chest indrawing, NO stridor	ፈጣን አተነፋፈስ (≥ 50 ትንፋሽ በደቂቃ)<br>Fast breathing<br>(≥50 breaths)	ዝቅተኛ የኦክስጅን መጠን (≤90%)<br>SP ≤90%	የሳንባ ምች እና ዝቅተኛ የኦክስጅን መጠን<br>PNEUMONIA with HYPOXIA	
• ሳል ወይም የመተንፈስ ችግር ያለው እና<br>• ስትራይደር ያሌለው ወይም<br>• የአደገኛ በሽታ ምልከቶች የሌለው ወይም<br>• የደረት መሰርጎድ የሌለው<br>History of cough or difficulty breathing<br>AND NO general danger signs, NO chest indrawing, NO stridor	ፈጣን ያልሆነ አተነፋፈስ (≤49 ትንፋሽ በደቂቃ)<br>Normal breathing (≤49 breaths)	ዝቅተኛ የኦክስጅን መጠን (≤90%)<br>SP ≤90%	ዝቅተኛ የኦክስጅን መጠን<br>(የሳንባ ምች ወይም በጣም ከባድ በሽታ የሌለው)<br>HYPOXIA<br>No signs of severe disease or fast breathing	በአፋጣኝ ኦክስጂን ወዳለበት ሆስፒታል ሪፈር ማድረግ<br>REFER URGENTLY to hospital with oxygen therapy
• ሳል ወይም የመተንፈስ ችግር ያለው እና<br>• ስትራይደር ያሌለው ወይም<br>• የአደገኛ በሽታ ምልከቶች የሌለው ወይም<br>• የደረት መሰርጎድ የሌለው<br>History of cough or difficulty breathing<br>AND NO general danger signs, NO chest indrawing, NO stridor	ፈጣን አተነፋፈስ (≥ 50 ትንፋሽ በደቂቃ)<br>Fast breathing<br>(≥50 breaths)	የኦክስጅን መጠን ዝቅተኛ አይደለም<br>(>90%)<br>SP >90%	የሳንባ ምች<br>PNEUMONIA	በአሞክሳስሊን ዲቲ በ ቀን 2 ጊዜ ለ5 ቀን ህክምና ስጪ<br>Treat with oral amoxicillin 2 times a day for 5 days
• ሳል ወይም የመተንፈስ ችግር ያለው እና<br>• ስትራይደር ያሌለው ወይም<br>• የአደገናኛ በሽታ ምልከቶች የሌለው ወይም<br>• የደረት መሰርጎድ የሌለው<br>History of cough, NO general danger signs, NO chest indrawing, NO stridor	ፈጣን ያልሆነ አተነፋፈስ (≤49 ትንፋሽ በደቂቃ)<br>Normal breathing<br>(≤49 breaths)	የኦክስጅን መጠን ዝቅተኛ አይደለም<br>(>90%)<br>SP >90%	ሳል ወይም ጉንፋን<br>COUGH or COLD	የቤት ውስጥ ክብካቤ እነድታደርግ ለእናትየው ምክር ስጪ<br>Advise mother to give homecare

12 to 59 ወራት በከባድ ህመም ምልክቶች፣ በኦክስጅን እና አተነፋፈስ መጠን መሰረት መሰየም

STEP 6: Classify child 12-59 months based on SpO2, RR results, and danger signs

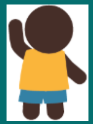

የከባድ በሽታ ምልክቶች<br>**Signs of illness 12 to 59 months**	የትንፋሽ መጠን<br>**RR**	የኦክስጅን መጠን<br>**SpO2**	ስያሜ<br>**Classification**	ህክምና<br>**Treatment**
• ሳል ወይም የመተንፈስ ችግር ያለው እና<br>• በተረጋጋ ልጅ ስትራይደር ያለው ወይም<br>• የአደገኛ በሽታ ምልክቶች ያለው ወይም<br>• የደረት መሰርጎድ ያለው<br>• History of cough or difficulty breathing, AND<br>• Stridor in calm child, OR<br>• Any general danger sign OR<br>• chest indrawing	ፈጣን አተነፋፈስ ≥40 ትንፋሽ በደቂቃ)<br>ወይንም<br>ፈጣን ያልሆነ አተነፋፈስ (≤39 ትንፋሽ በደቂቃ<br>Fast breathing<br>(≥40 breaths)<br>OR<br>Normal breathing (≤39 breaths)	የኦክስጅን መጠን ዝቅተኛ አይደለም (>90%)<br>SP >90%	ከባድ የሳንባ ምች ወይም በጣም ከባድ በሽታ<br>SEVERE PNEUMONIA<br>or VERY SEVERE DISEASE	የቅድመ ሪፈራል መድሀኒት መስጠትና በአፋጣኝ ኦክስጂን ወዳለበት ሆስፒታል ሪፈር ማድረግ<br>Give first dose of an appropriate antibiotic and REFER URGENTLY to hospital with oxygen therapy
		ዝቅተኛ የኦክስጅን መጠን<br>(≤90%)<br>SP ≤90%	ከባድ የሳንባ ምች ወይም በጣም ከባድ በሽታ እና ዝቅተኛ የኦክስጅን መጠን<br>SEVERE PNEUMONIA or VERY SEVERE DISEASE with HYPOXIA	
• ሳል ወይም የመተንፈስ ችግር ያለው እና<br>• ስትራይደር ያሌለው ወይም<br>• የአደገኛ በሽታ ምልክቶች የሌለው ወይም<br>• የደረት መሰርጎድ የሌለው<br>History of cough or difficulty breathing<br>AND NO general danger signs, NO chest indrawing, NO stridor	ፈጣን አተነፋፈስ ≥40 ትንፋሽ በደቂቃ)<br>Fast breathing<br>(≥40 breaths)	ዝቅተኛ የኦክስጅን መጠን<br>(≤90%)<br>SP ≤90%	PNEUMONIA with HYPOXIA	
• ሳል ወይም የመተንፈስ ችግር ያለው እና<br>• ስትራይደር ያሌለው ወይም<br>• የአደገኛ በሽታ ምልክቶች የሌለው ወይም<br>• የደረት መሰርጎድ የሌለው<br>History of cough or difficulty breathing<br>AND NO general danger signs, NO chest indrawing, NO stridor	ፈጣን ያልሆነ አተነፋፈስ (≤39 ትንፋሽ በደቂቃ<br>Normal breathing (≤39 breaths)	ዝቅተኛ የኦክስጅን መጠን (≤90%)<br>SP ≤90%	ዝቅተኛ የኦክስጅን መጠን<br>(የሳንባ ምች ወይም በጣም ከባድ በሽታ የሌለው)<br>HYPOXIA<br>No signs of severe disease or fast breathing	በአፋጣኝ ኦክስጂን ወዳለበት ሆስፒታል ሪፈር ማድረግ<br>REFER URGENTLY to hospital with oxygen therapy
• ሳል ወይም የመተንፈስ ችግር ያለው እና<br>• ስትራይደር ያሌለው ወይም<br>• የአደገኛ በሽታ ምልክቶች የሌለው ወይም<br>• የደረት መሰርጎድ የሌለው<br>History of cough or difficulty breathing<br>AND NO general danger signs, NO chest indrawing, NO stridor	ፈጣን አተነፋፈስ ≥40 ትንፋሽ በደቂቃ)<br>Fast breathing<br>(≥40 breaths)	የኦክስጅን መጠን ዝቅተኛ አይደለም<br>(>90%)<br>SP >90%	የሳንባ ምች<br>PNEUMONIA	በአሞክሳሰሊን ዲቲ በ ቀን 2 ጊዜ ለ5 ቀን ህክምና ስጪ<br>Treat with oral amoxicillin 2 times a day for 5 days
• ሳል ወይም የመተንፈስ ችግር ያለው እና<br>• ስትራይደር ያሌለው ወይም<br>• የአደገናኛ በሽታ ምልክቶች የሌለው ወይም<br>• የደረት መሰርጎድ የሌለው<br>History of cough, NO general danger signs, NO chest indrawing, NO stridor	ፈጣን ያልሆነ አተነፋፈስ (≤39 ትንፋሽ በደቂቃ<br>Normal breathing<br>(≤39 breaths)	የኦክስጅን መጠን ዝቅተኛ አይደለም (>90%)<br>SP >90%	ሳል ወይም ጉንፋን<br>COUGH or COLD	በአሞክሳሰሊን በ ቀን 2 ጊዜ ለ5 ቀን ህክምና ስጪ<br>Advise mother to give homecare

ጥቅም ላይ የዋለውን መሳሪያ መመዝገብ

STEP 7: Record which device used

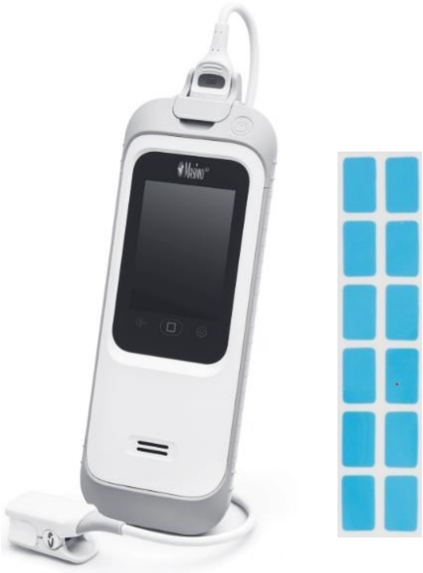

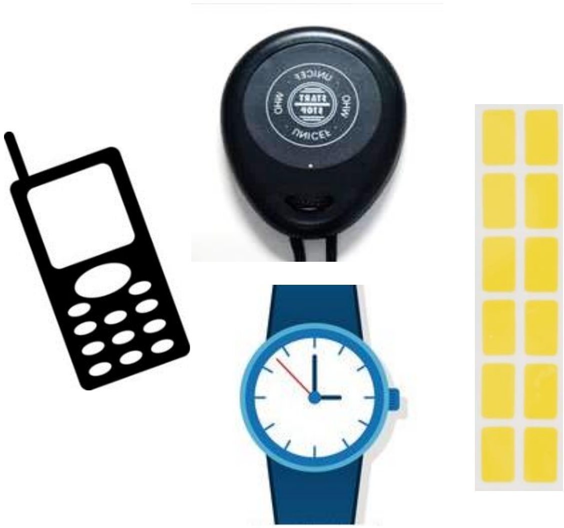

የራድ ጂ መሳሪያን ከተጠቀምሽ ሰማያዊ ስቲከር በመዝገብሽ ላይ ለጥፊ

Stick a blue sticker in your logbook if you used the Rad-G device

ከራድ ጂ መሳሪያ ውጪ ከተጠቀምሽ ቢጫ ስቲከር በመዝገብሽ ላይ ለጥፊ

Stick a yellow sticker in your logbook if you used your usual device (ARI timer, phone, or watch)

malaria consortium
disease control, better health

ህፃኑ ሪፈር ካልተደረገ ቀሪ ምርመራዎችን አጠናቂ

If child was not referred, complete remainder of child assessment

ከ1-7 ድረስ ያሉ ተግባራትን ካጠናቀቅሽ በyላ ምርመራሽን በአይሲሲኤም መሰረት ቀጥይ

Once you have completed STEPS 1-6, please continue child assessment as usual

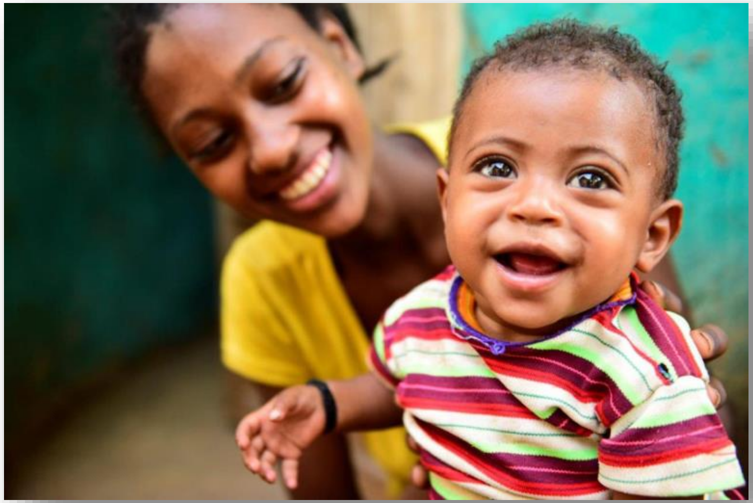

ከ0-2 ወራት ያላቸውን ጨቅላዎች ለበጣም ከባድ በሽታ በአይሲሲኤም መመሪያ መሰረት ማከም

STEP 8: Treat children 0 to 2 months with very severe disease according to national guidelines

ዕድሜ

ከ 0 – 2 ወራት

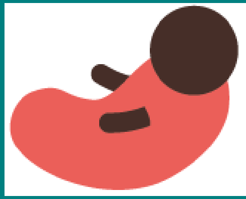

0 to <2 months

አሞክሳሰሊን Amoxicillin

- በጣም ከባድ በሽታ ላለዉ ጨቅላዎች የሚሰጥ ህክምና ... በቀን ሁለት ግዜ ለ7 ቀናት ስጪ

  For very severe disease, give 2 times/day for 7 days
- ለዉስን የባክተሪያል እንፌክሽን የሚሰጥ ህክምና ... በቀን ሁለት ጊዜ በየቀኑ ለ 5 ቀናት ስጪ

  For local bacterial infection give 2 times/day for 5 days
- ለቅድመ ሪፈራል ... ከሪፈራል በፊት አንድ ዶዝ ስጪ

  For pre-referral treatment give 1st dose

ክብደት<br><br>Weight	በቅፅበት ማ*ሚ ኪኒን ባለ 125 ሚሊ ግራም ይሰጣል<br><br>Dispersed tablet 125mg	በቅፅበት ማ*ሚ ኪኒን ባለ 250 ሚሊ ግራም ይሰጣል<br><br>Dispersed tablet 250mg	ባለ 125 ሚሊ ግራም ሽሮፕ በ 5ሚሊ መጠን ይሰጣል<br><br>Syrup 125mg / 5ml
ከ 2000 ግራም በታች<br><br>< 2,000g	ግማሽ እንክብል<br><br>½ tablet	ሩብ እንክብል<br><br>¼ tablet	2.5 ሚሊ ሊትር<br><br>2.5ml
ከ2000 እስከ 4500 ግራም<br><br>2,000g to < 4,500g	አንድ ሙሉ እንክብል<br><br>1 tablet	ግማሽ እንክብል<br><br>½ tablet	5 ሚሊ ሊትር<br><br>5ml

malaria consortium
disease control, better health

ከ0-2 ወራት ያላቸውን ጨቅላዎች ለበጣም ከባድ በሽታ በአይሲሲኤም መመሪያ መሰረት ማከም

STEP 8: Treat children 0 to 2 months with very severe disease according to national guidelines

ዕድሜ

ከ 0 – 2 ወራት

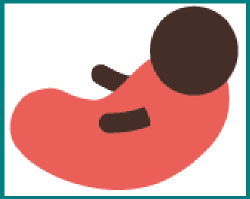

0 to <2 months

ጀንታማይስን IM Gentamicin:

- በጣም ከባድ በሽታ ላለዉ ጨቅለ የሚሰጥ ህክምና … በቀን አንድ ግዜ ለ7 ቀናት ስጪ

  For very severe disease once /day for 7 days
- በጣም ከባድ በሽታ ላለዉ ጨቅላ የሚሰጥ ህክምና ክብደታቸዉ ከ2000 ግራም በታች በየ48ሰዓቱ አንዴ ለ4ቀናት ስጪ ፡፡

  For very severe disease for less than 2,000g every 48 hours once for 4 days
- ለቅድመ ሪፈራል … ከሪፈራል በፊት አንድ ዶዝ ስጪ

  For pre-referral treatment give 1st dose

ክብደት<br>Weight	ጀንታማይሲን 20ሚ.ግ/2ሲሲ<br>Gentamicin 20mg /2cc	ጀንታማይሲን 80ሚ.ግ/2ሲሲ<br>Gentamicin 80mg/ 2cc
ከ 2000 ግራም በታች<br>< 2,000g	1ሲሲ በየ48 ሰዓት /<br>1 cc every 48 hours	0.3 ሲሲ በየ48 ሰዓት<br>0.3 cc every 48 hours
ከ2000 እስከ 2500 ግራም<br>2,000g to < 2,500g	1 ሲሲ በቀን /<br>1cc daily	0.3 ሲሲ በቀን /<br>0.3 cc daily
2500 እስከ 3500 ግራም<br>2,500 to < 3,500 g	1.4 ሲሲ በቀን /<br>1.4 cc daily	0.4 ሲሲ በቀን /<br>0.4 cc daily
3500 እስከ 4500 ግራም<br>3,500 to < 4,500g	2ሲሲበቀን /<br>2cc daily	0.5 ሲሲ በቀን /<br>0.5 cc daily

malaria consortium
disease control, better health

ከ2-59 ወራት ያላቸውን ህፃናት ለሳንባ ምች በአይሲሲኤም መመሪያ መሰረት ማከም

STEP 8: Treat children 2 to 59 months with pneumonia according to national guidelines

ዕድሜ ከ 2 – 59 ወራት

For 2-59 months

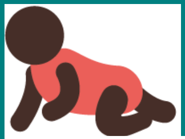

2 to <12 months

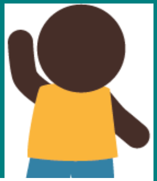

12 to 59 months

አሞክሳስሊን በቀን 2 ጊዜ ለ 5 ቀን ስጪ

Oral amoxicillin 2 times a day for 5 days

ዕድሜ እና ክብደት<br>Age and weight	ባለ 125 ሚሊ ግራም ይሰጣል<br>Dispersed 125mg	ባለ 250 ሚሊ ግራም ይሰጣል<br>Dispersed 250mg	ባለ 125 ሚሊ ግራም ሽሮፕ በ 5ሚሊ መጠን ይሰጣል<br>Syrup 125mg / 5ml
ከ2 እስከ 12 ወራት (ከ4 እስከ 10 ኪሎ ግራም በታች)<br>2 to < 12 months (4 to < 10kg)	2 እንክብል<br>2 tablets	1 እንክብል<br>1 tablets	5 ሚሊ<br>5ml
ከ12 እስከ 3 ዓመት (ከ10 እስከ 14 ኪሎ ግራም በታች)<br>12 months to <3 years (10 to < 14kg)	4 እንክብል<br>4 tablets	2 እንክብል<br>2 tablets	10 ሚሊ<br>10 ml
ከ 3 ዓመት እስከ 5 ዓመት / 14 – 19 ኪ.ግ/<br>3 years to < 5 years (14 to < 19kg)	6 እንክብል<br>6 tablets	3 እንክብል<br>3 tablets	15 ሚሊ<br>15ml

ስለሳንባ ምች መከላከያ መንገድ ማስተማር

STEP 9: Give pneumonia prevention messages

ለመጀመሪያዎቹ 6 ወራት ህፃኑን
የእናት ጡት ወተት ብቻ ይመግቡ

Feed your child only breast milk for the first 6 months of life

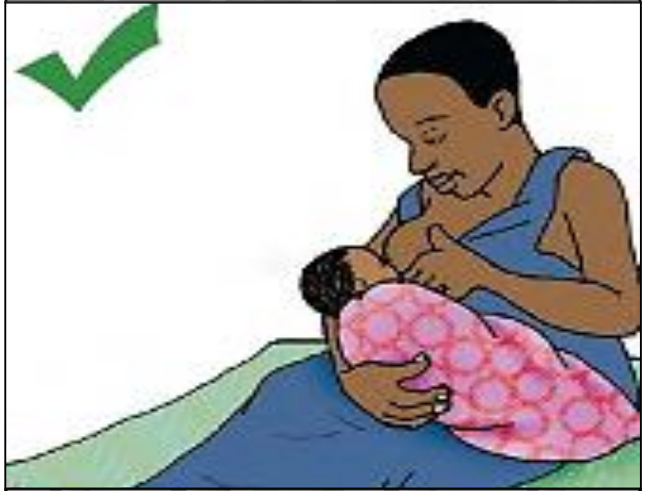

ህፃናትን እስከ 2 ዓመትና ከዚያም
በላይ የእናት ጡት ወተት
እንዲጠቡ አድርጉ

Breast feed children for 2 years or more

ልጆችን በግዜ አስከትቡ

Take children for immunizations

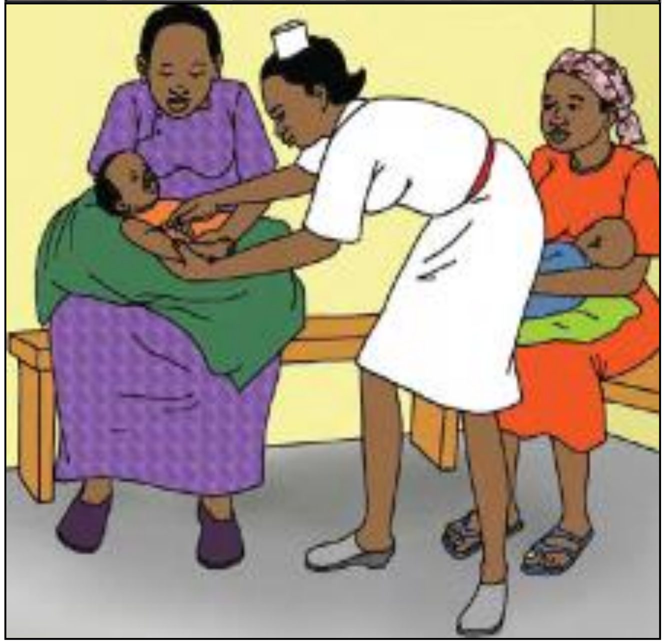

malaria consortium
disease control, better health

ስለሳንባ ምች መከላከያ መንገድ ማስተማር

STEP 9: Give pneumonia prevention messages

ልጆች የተመጣጠነና በቂ ምግብ እንዲመገቡ ያድርጉ

Give children enough and different nutritious foods to eat

ህፃናትና ልጆች ለቤት ውስጥ የአየር ብክለት እንዳይጋለጡ ያድርጉ

Keep babies and children away from smoky rooms
Cook in a well ventilated area.

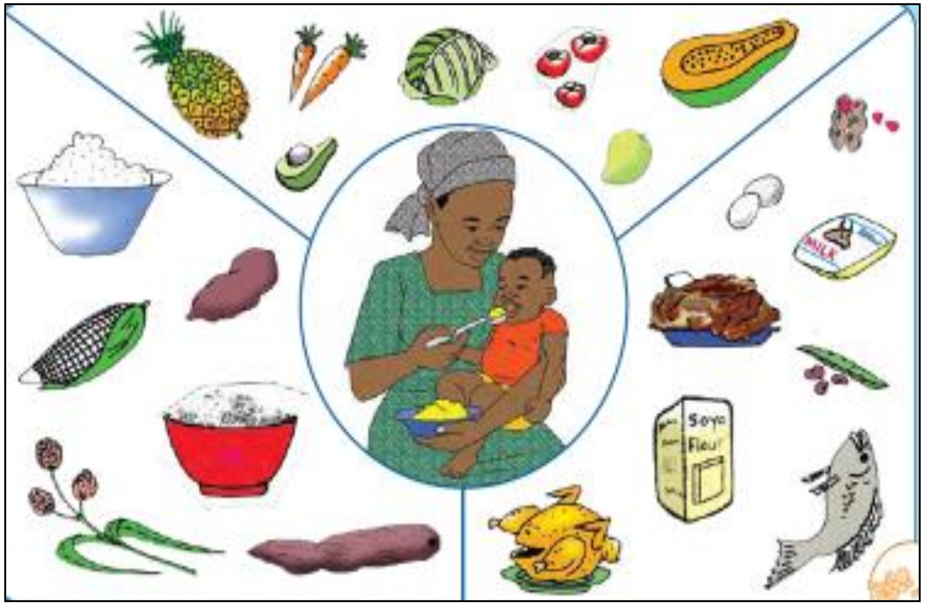

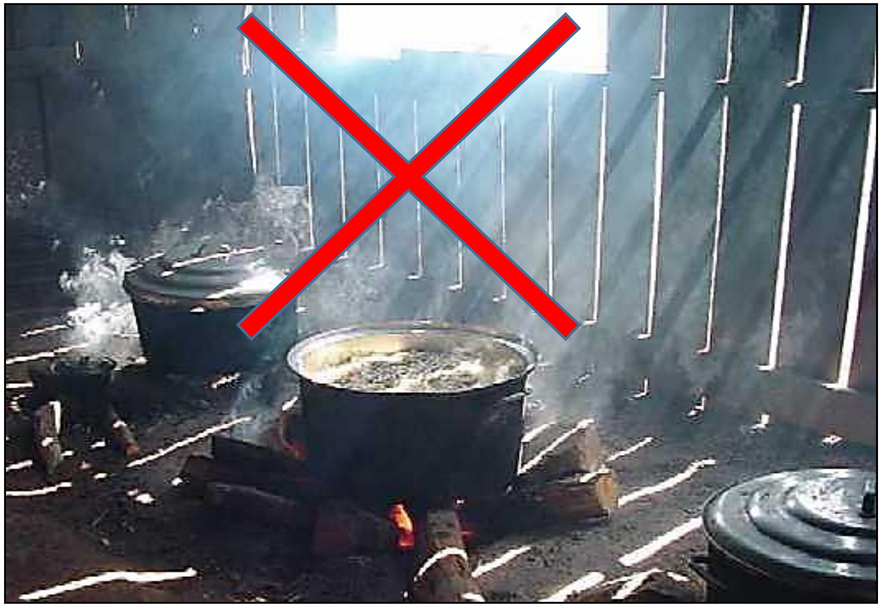

malaria consortium
disease control, better health

አስፈላጊ አድራሻዎች - CONTACT CARD

የአጣዳፊ የመተንፈሻ አካላት በሽታ ምርመራ አጋዥ መሣርያ ጥናት ዓላማ የጤና ኤክስቴንሽን ሰራተኞች እና የጤና ባለሙያዎች ከ5 ዓመት በታች ያሉ ሕፃናትን ላይ አተነፋፈስ መቁጠሪያ አጋዥ መሳርያ አጠቃቀምን ለመመዘን ነው::

The Acute Respiratory Infection Diagnostic Aids (ARIDA) project aims to evaluate community health workers' and facility health workers' use of respiratory rate counting aids on children under five in Ethiopia.

ከጥናቱ ጋር በተያያዘ ምንም ዓይነት ችግር ቢያጋጥምዎት ለሚከተሉት ባልደረቦቻችን በአድራሻቸዉ ያሳዉቁ :

Should you experience any issue related to the study, please contact the following people to report it:

- በመሳሪያው ላይ ለሚታዩ ችግሮች: ለ እስክንድር ጎሹ, የመስክ አስተባባሪ, e.goshu@malariaconsortium.org +2510911825630

  Device fault: Eskinder Goshu, MC Site Coordinator, e.goshu@malariaconsortium.org, +2510911825630

- ከመሳሪያው ጋር ተጓዳኝነት ያለቸው ከባድ የህመም ክስተቶች: ለ ተድላ ሃብቴ, የፕሮጀክቱ ሥራአስኪያጅ +251 931404500 t.habte@malariaconsortium.org

  Severe adverse event: Tedila Habte, MC Project Manager, t.habte@malariaconsortium.org, +251 931404500

- ለመረጃ አሰባሰብን በተመለከተ: ለየኔዓለም ረታ, ዳታ ማናጀር miyeyeni@gmail.com, +251911973794

  Data enquiry: Yene Rata, MC Data Manager, miyeyeni@gmail.com, +251911973794

- በአጠቃላይ ጥናቱን በተመለከተ ለሚኖሩ ጥያቄዎች:ለ እስክንድር ጎሹ, የመስክ አስተባባሪ, e.goshu@malariaconsortium.org +2510911825630

  General study enquiry: Eskinder Goshu, MC Technical Officer, e.goshu@malariaconsortium.org, +2510911825630

- ለማናቸውም አግባብነት የሌላቸው አሰራር ስህተቶችን በተመለከተ : ለ ዶ/ር አጎናፍር ተካልኝ, ካንትሪ ዳይሬክተር a.tekalegne@malariaconsortium.org +251 (0)118963443፣ ኬቨን ቤከር, ፕሮግራም አስተባባሪ k.baker@malariaconsortium.org +44 (0)20 7549 0270

  Abuse: Agonafer Tekalegne, MC Country Director, a.tekalegne@malariaconsortium.org, +251 (0)118963443

  Kevin Baker, MC Programme Coordinator, k.baker@malariaconsortium.org, +44 (0)20 7549 0270

"la Caixa" Banking Foundation

unicef

malaria consortium
disease control, better health
