# Supplementary material for: Automated Respiratory Rate Counter to Assess Children for Symptoms of Pneumonia: Protocol for Cross-Sectional Usability and Acceptability Studies in Ethiopia and Nepal
Source: JMIR Res Protoc. 2020 Mar 30;9(3):e14405. doi: 10.2196/14405 (PMC7154937; doi:10.2196/14405)
Supplement: Multimedia Appendix 2 [file resprot_v9i3e14405_app2.docx]

**Multimedia Appendix 2. Quality Assurance Assessment Form.**

Date __________________________

Name and Study ____________

| **Step No. & Name** | **Description of Procedure** | **Person(s) Responsible by Title** | **Activity/Documents involved** | **Score (1=Fail to 5=excellent) + Comments** |
| --- | --- | --- | --- | --- |
| **Pre-assessment** |  |  |  |  |
| Training | - CHWs will have completed training and understand their role and are performing as required | CHWs | Observation |  |
| Staff management | - Research team are functioning well and adhering to assigned roles and responsibilities | RAs | Observation |  |
|  | - Daily meetings are being held to agree activities/data management and other activities as required | RAs | Observation |  |
| Site management | - Research site is well set-up and correctly equipped to conduct the study as per protocol | PM | Observation |  |
| **Protocol** |  |  |  |  |
| 1. Screening | - Research assistant screen children correctly | RAs | Screening Form |  |
| 1. Consent/caregiver engagement | - Caregivers will have been consented correctly to take part in evaluation | RAs | Consent Form |  |
|  | - Caregivers are not waiting too long or being treated unfairly | RAs | Observation |  |
| 1. Protocol compliance | - RAs are completely adhering to the protocol when conducting the assessments for the acceptability study (observe 2 full evaluations) | RAs | Observation |  |
| 1. Data capture | - RAs are using tablets correctly to capture data – independently entering data and completing the whole checklist correctly | RAs | Observation |  |
|  | - Tablets are being maintained well – (check app number and record as well as battery levels) | Data manager (DM) | Observation |  |
| **Post Assessment** |  |  |  |  |
| Data management | - All data is checked daily by the DM and data cleaning, eg checking consent withdrawals do not have relevant forms completed, is conducted with relevant staff - RAs | DM/ RAs | Data form review X 5 |  |
|  | - Weekly reporting and updating of data tables to be conducted by DM | DM | Observation |  |
|  | - Data validation is to be undertaken in country daily | PM |  |  |
|  | - A sample of data has been sent to the statistician for review. | Project manager |  |  |
| Query Process | - Queries are routinely logged and resolved. | ? |  |  |
| Devices Collected | - All study devices have been collected. | ? |  |  |

**Average score:**

**Signed:**
